# Making the continuum of care work for mothers and infants: Does gender equity matter? Findings from a quasi-experimental study in Bihar, India

**DOI:** 10.1371/journal.pone.0171002

**Published:** 2017-02-01

**Authors:** Lotus McDougal, Yamini Atmavilas, Katherine Hay, Jay G. Silverman, Usha K. Tarigopula, Anita Raj

**Affiliations:** 1 Center on Gender Equity and Health, Division of Global Public Health, Department of Medicine, University of California, San Diego School of Medicine, San Diego, California, United States of America; 2 Bill and Melinda Gates Foundation, New Delhi, India; National Institute of Health, ITALY

## Abstract

**Background:**

Improvements in continuum of care (CoC) utilization are needed to address inadequate reductions in neonatal and infant mortality in India and elsewhere. This study examines the effect of Ananya, a health system training and community outreach intervention, on reproductive, maternal and newborn health continuum of care (RMNH CoC) utilization in Bihar, India, and explores whether that effect is moderated by gender equity factors (child marriage, restricted mobility and low decision-making control).

**Methods:**

A two-armed quasi-experimental design compared districts in Bihar that did/did not implement Ananya. Cross-sections of married women aged 15–49 with a 0–5 month old child were surveyed at baseline and two year follow-up (baseline n = 7191 and follow-up n = 6143; response rates 88.9% and 90.7%, respectively). Difference-in-difference analyses assessed program impact on RMNH CoC co-coverage, defined by 9 health services/behaviors for the index pregnancy (e.g., antenatal care, skin-to-skin care). Three-way interactions assessed gender equity as a moderator of Ananya’s impact.

**Findings:**

Participants reported low RMNH CoC co-coverage at baseline (on average 3.2 and 3.0 of the 9 RMNH services/behaviors for Ananya and control groups, respectively). The Ananya group showed a significantly greater increase in RMNH CoC co-coverage (.41 services) compared with the control group over time (p<0.001), with the primary drivers being increases in clean cord care, skin-to-skin care and postpartum contraceptive use. Gender equity interaction analyses revealed diminished intervention effects on antenatal care, skilled birth attendance and exclusive breastfeeding for women married as minors.

**Conclusion:**

Ananya improved RMNH CoC co-coverage among these recent mothers, largely through positive health behavior changes. Child marriage attenuated Ananya’s impact on utilization of key health services and behaviors. Supporting the health system with training and community outreach can be beneficial to RMNH CoC utilization; additional support is needed to adequately address the unique issues faced by women married as minors.

## Introduction

Despite substantial global attention and resources devoted to maternal and child health over the past decade, annually more than 300,000 women die as a result of pregnancy and childbirth, and more than 2.6 million children die within their first month of life [1–4]. Great acceleration is needed to meet the ambitious goals set forth in the Sustainable Development Goals, including the reduction of the global maternal mortality ratio (MMR) to under 70 per 100,000 live births (current level MMR is 216/100,000) and the reduction of neonatal mortality rate (NMR) to 12 per 1000 live births or lower (current level NMR is 19/1000) [3, 4]. The model being widely supported to improve these outcomes is the continuum of care (CoC), in which quality, effective, evidence-based health services are provided as an integrated stream across levels and places of service delivery and life stages for women and children [2, 5, 6]. While coverage along the CoC has improved globally, it is still low, with little improvement seen over time for many key reproductive and maternal health services, including four or more antenatal care visits, skilled attendant at delivery, exclusive breastfeeding and met need for family planning [2]. Further, despite widespread advocacy for the CoC [5–7], much remains unknown as to how to effectively increase utilization across the entire continuum [8–10]. No rigorously evaluated intervention research has documented significant improvements in overall CoC utilization, though there is a large evidence base for the efficacy of the individual interventions that comprise it, and for the importance of linkages across those services [7, 11–14].

India is a critical setting in which to implement and evaluate programs designed to improve maternal and child health, as it accounts for some of the greatest numbers of maternal and child deaths in the world [3, 4]. Subnationally, there is enormous variability in health outcomes across Indian states. Bihar is one of the most populous, poorest and lowest-performing states in the nation, with great need for improvements in health services and outcomes [15]. More than 50% of adult women are illiterate, with fewer than one quarter of women receiving ten or more years of education [16]. Infant mortality, maternal mortality, total fertility are all above the national average, and gender equity is compromised, with 39% of women married at 18 years of age or less [15, 16]. Modern contraceptive use in married women has actually decreased over the past ten years, from 29% in 2006 to 23% (nearly 90% of which is female sterilization) [16]. Additionally, public health care access in Bihar is inadequate; the state has only 60% of the necessary primary health centers, and 9% of the required community health centers [15]. More than half of all institutional births take place in private, rather than public, facilities [16]. Reasons given for not seeking care from government health facilities include the poor quality of care received therein, and lack of nearby facilities [17]. Gender equity factors, including early age at marriage and childbirth, as well as restricted mobility and decision-making control, have also been implicated in low utilization of services and poorer maternal and child health outcomes, in India and Bihar [16, 18–23].

In response to these needs, the Bill and Melinda Gates Foundation partnered with the Government of Bihar in 2011 to implement a set of innovations under the Ananya program in 8 districts of Bihar [24]. This program uses supply- and demand-side interventions to improve reproductive, maternal, newborn and child health (RMNCH) services and outcomes, focusing in particular on interventions delivered through frontline workers (FLWs), i.e., those health workers engaging in community outreach and connection to women in communities and households. Improving outreach services provided by FLWs was prioritized (particularly through home-visits) as a means of identifying and engaging women not currently using the health system, improving retention along the continuum of care, and helping to address ongoing disparities in health service utilization and outcomes in Bihar, particularly for the poor, rural, less educated and religious and caste minorities. Initial evaluation of this program identified improvements in select reproductive, maternal and neonatal health (RMNH) behaviors, such as clean cord care and post-partum contraceptive use, but results were not consistent across social equity groups, with greater improvements seen within some socially marginalized groups [25]. This variability is particularly important given lower coverage of health service utilization among poorer, more rural and less educated women [2, 26, 27]. RMNH CoC co-coverage was not evaluated in the original study.

This paper assesses whether the Ananya program was able to increase overall RMNH CoC among women who gave birth in the previous six months. We also assess whether any observed impact of Ananya on RMNH CoC utilization in these recent mothers is moderated by gender equity factors, including young maternal age at marriage, decision-making control and freedom of mobility, as prior research from India documents that these factors compromise RMNH practices [18–20, 22, 23]. Findings from this work can not only inform RMNH programming in Bihar and similar settings, but also contribute to the growing understanding of how to improve CoC co-coverage.

## Methods

### Intervention

The Ananya program was developed and implemented via a partnership between the Government of Bihar and the Bill and Melinda Gates Foundation as a means of testing high impact solutions for improving reproductive, maternal, newborn and child health services and outcomes in 8 districts of Bihar [24]. Ananya was designed to improve supply of and demand for services delivered during the critical 1,000 day window from conception to a child’s second birthday [28], in particular focusing on health behaviors and service utilization in the final trimester of pregnancy and early postpartum period. The program was implemented in eight of Bihar’s 38 districts (Begusarai, Gopalganj, Khagaria, Paschim Champaran, Patna, Purba Champaran, Saharsa and Samastipur) and was designed as a series of supply- and demand-side interventions. Interventions have been described elsewhere [24], and included improving quality and quantity of outreach services to strengthen access to the health system, improving quality of services, and mobilizing communities to improve health behaviors. Activities to strengthen outreach involved training, mobilizing, and monitoring of government FLWs (including accredited social health activists[ASHAs], auxiliary nurse midwives[ANMs], and anganwadi [social service] workers) and empowering them with job aids and tools to increase quantity, quality, and ultimately, effectiveness of home visits for RMNH screenings and services to increase demand for services. Tools included the *mobile kunji*, an interactive voice response-based mobile service and a printed deck of cards covering messages related to 10 life-saving RNMCH behaviors to help enhance FLWs’ counseling of families, and *mobile academy*, a mobile training course for FLWs to expand and refresh their knowledge of life-saving RMNCH behaviors.

### Study design, sample and procedure

A two-armed quasi-experimental design study compared women from the eight districts in which the Ananya program was implemented to those from the remaining 30 districts who received the existing standard of care. To assess the impact of the Ananya program, state-wide household surveys were conducted January-April 2012 (baseline) and January-April 2014 (follow-up) using two-stage population proportionate to size stratified sampling without replacement [25]. Strata were selected by district and urban/rural blocks within each district, and clusters were selected at the community level (villages for rural areas, urban blocks for urban areas). In large rural villages (≥150 households), there was a third stage of sampling in which villages were subdivided into equal groups of 75–150 households. In each cluster, all women who had given birth in the 12 months prior to the date of interview were eligible to be interviewed. Trained female field staff approached households identified as having an eligible women resident and invited the eligible woman to participate in the survey. Those willing and able to participate provided written informed consent and were surveyed in a private location by the staff member. Data were collected electronically and uploaded daily for data management and review.

The baseline survey included interviews with 13,069 women (88.9% response rate), and the follow-up survey included 12,015 women (90.7% response rate). As this paper focuses on reproductive, maternal and neonatal health services and behaviors, the analytic sample was further restricted, with inclusion criteria as follows: women aged 15–49 years surveyed by Ananya baseline or follow-up surveys who were currently married, not pregnant, had given birth to a living, singleton child in the past six months (“recent mothers”), and had responses for all variables used in this analysis (n = 13,334; 7,191 baseline and 6,143 follow-up). Women whose most recent birth was greater than six months prior to interview were excluded from this analysis.

### Measures

The primary dependent variable was continuum of care co-coverage, or the number of a defined list of evidence-based interventions received by a mother-child dyad [10]; components of this list have not yet been standardized across the global maternal and child health community [10, 14, 29, 30]. This outcome focuses on interventions/health behaviors during the pregnancy and early postpartum window, a subset of the 1,000 day window of opportunity for health improvement that is a particular focus of the Ananya program, and that has been identified as a period of high inequity [27]. Interventions targeting children aged 6–23 months are of great importance to child health and were components of Ananya, but focus on a different subset of children than covered in this sample, and are thus outside the scope of this analysis. In this study, co-coverage is defined as the number of services or health behaviors (0–9) received related to the index pregnancy/delivery. The components are: at least four antenatal care visits, skilled birth attendant at delivery, nothing applied to cord/umbilicus, skin-to-skin care (child placed unclothed on mother’s chest/abdomen in skin-to-skin contact), first bath delayed by two or more days, breastfed child within one hour of birth, postnatal care home visit from a FLW for mother or baby within 2 days of birth, child exclusively breastfed and postpartum contraception (current use of modern contraception, defined as female or male sterilization, pill, IUD, injectables or condoms).

The independent variable of focus was the Ananya intervention, specifically, whether or not respondents lived in Ananya program districts. Covariates assessed a range of demographic, social equity and gender equity factors. Background demographic and social equity metrics included age of the respondent at interview (15–19, 20–24, 25–29, 30–34, ≥35), wealth quartile (derived from a principal components analysis of floor, roof and wall materials, source of drinking water, type of toilet, type of cooking fuel and number of household members per sleeping room), education level for both the respondent and her husband (none, primary [1–8 years] or secondary or higher [≥9 years]), caste/religion (SC/ST, Muslim, neither), gender of the focal child, parity (1, 2, ≥3 births) and whether or not the respondent received at least two FLW visits in the last trimester of pregnancy. Gender equity measures included age of the mother at marriage (<18, ≥18), as well as three-item measures of decision-making control and social mobility derived from the NFHS-3 [17]. Decision-making control assessed whether the respondent was always involved (either solely, or in partnership with others) in making decisions about her own health care, decisions about her child’s health care, and decisions about major household purchases vs. excluded from one or more decision. Mobility assessed whether a woman was allowed to the market, to the health facility, and to places outside her village/community by herself, versus being restricted from independent travel to any of the three locations.

### Analysis

Basic frequencies were calculated for background demographic, social equity and gender equity characteristics, and RMNH CoC components for Ananya and non-Ananya districts, and compared at baseline and follow-up using survey-adjusted unpaired t-tests. To assess the effects of the Ananya program on continuum of care coverage for individual women and their index children, we employed a difference-in-differences approach, in which models included dummy variables for time (0 for baseline, 1 for follow-up) to account for secular changes over time, and treatment (0 for non-Ananya districts, 1 for Ananya districts). The interaction of time and treatment gives the change in outcome attributable to the Ananya program. Unadjusted difference-in-difference estimates for each RMNH CoC component were assessed using logistic regression models. A multivariate linear model, adjusting for all aforementioned covariates, was used to compare CoC co-coverage in Ananya / non-Ananya districts from baseline to follow-up. Program impact on each of the nine RMNH CoC components that make up the primary outcome was assessed using individual multivariate logistic models for each component. Finally, the influence of gender equity factors (age at marriage, decision-making control and mobility) on program impact was assessed for CoC co-coverage (using multivariate linear regression) and for each of the nine component outcomes (using multivariate logistic regression) using three-way interaction terms of time x treatment x each gender equity factor. All analyses were performed using Stata 13 (StataCorp, College Station, TX).

### Ethics

Ethical approval for the original study was provided by India’s Health Ministry Screening Committee. Approval for this analysis was provided by the University of California, San Diego.

## Results

Participants were predominantly aged 20–24 years and had no formal education (Table 1). Women had similar characteristics across Ananya and non-Ananya districts at both baseline and follow-up, though at both timepoints, women living in Ananya districts tended to have higher numbers of births (women with 3 or more births = 45.5% vs. 39.7% at baseline, p = 0.004; 43.4% vs. 39.1% at follow-up, p = 0.04). At follow-up, both women and their husbands had a higher prevalence of primary education in Ananya areas vs. non-Ananya areas (28.2% vs. 24.4% for women, p = 0.046; 37.4% vs. 32.2% for husbands, p = 0.01), though secondary education in Ananya vs. non-Ananya areas was less prevalent for husbands, and marginally less prevalent for women (29.5% vs. 35.5% for husbands, p = 0.02, 18.6% vs. 22.5% for women, p = 0.06). At follow-up, a greater proportion of women in Ananya areas reported having received at least two FLW visits, a focus of the Ananya intervention, in their last trimester of pregnancy (37.7% vs. 29.2%, p = 0.01). In terms of gender equity among these recent mothers, while there were no significant differences between Ananya and non-Ananya areas at baseline, at follow-up, Ananya areas had significantly more early marriage (51.3% vs. 42.0%, p<0.0001), and more compromised decision-making (excluded from at least one decision: 63.1% vs. 51.6%, p<0.0001).

**Table 1:** Background characteristics of the sample, stratified by time and intervention group (n = 13,334).

	Baseline					Follow-up				
	Non-Ananya		Ananya			Non-Ananya		Ananya		
	Unwtd. N	% (95% CI)	Unwtd. N	% (95% CI)	p-value	Unwtd. N	% (95% CI)	Unwtd. N	% (95% CI)	p-value
Total	5,503	73.3 (69.9–76.4)	1,688	26.7 (23.6–30.1)	-	4,602	77.0 (73.9–79.8)	1,541	23.0 (20.2–26.1)	-
**Demographic and social equity**										
Age at interview										
15–19	201	4.1 (3.3–5.1)	58	2.9 (2.1–4.0)	0.08	161	4.4 (3.4–5.6)	77	5.8 (4.3–7.7)	0.18
20–24	2,166	38.6 (36.5–40.8)	682	39.9 (36.4–43.5)	0.53	2,024	43.8 (41.6–46.0)	646	40.8 (37.4–44.4)	0.17
25–29	2,051	37.1 (35.2–39.1)	618	37.5 (33.9–41.2)	0.86	1,734	36.5 (34.2–38.8)	548	36.1 (32.1–40.2)	0.86
30–34	719	12.8 (11.6–14.2)	236	14.2 (12.2–16.4)	0.30	517	11.3 (10.1–12.6)	191	12.5 (10.7–14.5)	0.33
35+	366	7.3 (6.3–8.5)	94	5.5 (4.1–7.4)	0.07	166	4.0 (3.2–5.0)	79	4.9 (3.4–6.9)	0.39
Wealth quartile										
1 (lowest)	1,387	27.1 (24.1–30.2)	427	22.4 (18.5–26.8)	0.07	1,308	29.9 (27.2–32.7)	432	28.8 (24.1–34.0)	0.70
2	1,311	24.5 (22.6–26.6)	390	24.6 (21.2–28.4)	0.98	950	20.5 (18.6–22.5)	310	20.6 (17.4–24.3)	0.94
3	1,346	25.2 (23.0–27.6)	427	27.5 (22.9–32.7)	0.41	1,113	25.2 (23.2–27.3)	368	23.8 (21.1–26.8)	0.46
4 (highest)	1,459	23.2 (20.8–25.7)	444	25.5 (21.6–29.9)	0.33	1,231	24.5 (22.1–27.0)	431	26.7 (22.3–31.7)	0.40
Education										
None	3,252	61.1 (58.5–63.6)	1,040	60.7 (56.9–64.5)	0.88	2,345	53.1 (50.0–56.2)	811	53.2 (49.0–57.4)	0.97
Primary	1,281	23.0 (21.2–25.0)	372	23.4 (20.4–26.6)	0.86	1,156	24.4 (22.4–26.5)	428	28.2 (25.1–31.4)	0.046
Secondary or higher	970	15.9 (14.2–17.8)	276	15.9 (13.2–19.1)	0.99	1,101	22.5 (20.3–24.9)	302	18.6 (15.3–22.3)	0.06
Husband’s education										
None	1,932	36.6 (34.0–39.4)	586	34.4 (29.1–40.0)	0.47	1,405	32.3 (29.5–35.1)	495	33.1 (28.8–37.7)	0.76
Primary	1,786	33.4 (31.2–35.7)	592	37.2 (33.1–41.6)	0.12	1,503	32.2 (30.1–34.4)	553	37.4 (34.5–40.5)	0.01
Secondary or higher	1,785	30.0 (27.8–32.3)	510	28.4 (24.8–32.3)	0.48	1,694	35.5 (32.8–38.4)	493	29.5 (25.4–33.9)	0.02
Scheduled caste/scheduled tribe/ Muslim										
Neither SC/ST nor Muslim	3,098	54.2 (50.4–57.8)	886	56.9 (49.1–64.3)	0.53	2,689	56.8 (52.9–60.6)	780	52.5 (46.3–58.6)	0.24
SCST	1,484	27.7 (24.6–31.1)	439	24.1 (18.9–30.2)	0.28	1,210	26.5 (23.2–30.1)	475	31.5 (26.7–36.7)	0.11
Muslim	921	18.1 (14.6–22.3)	363	19.0 (13.6–26.0)	0.80	703	16.7 (13.5–20.5)	286	16.1 (11.5–21.9)	0.84
Gender of focal child					0.59					0.83
Male	2,800	51.5 (49.5–53.6)	866	52.8 (48.8–56.7)		2,445	52.8 (50.8–54.8)	832	53.3 (49.8–56.8)	
Female	2,703	48.5 (46.4–50.5)	822	47.2 (43.3–51.2)		2,157	47.2 (45.2–49.2)	709	46.7 (43.2–50.2)	
Parity										
1 birth	1,790	31.9 (29.8–34.0)	513	29.3 (25.9–33.0)	0.22	1,439	32.2 (30.0–34.4)	437	28.8 (25.7–32.1)	0.08
2 births	1,530	28.4 (26.7–30.1)	430	25.2 (22.0–28.7)	0.10	1,365	28.7 (26.7–30.7)	431	27.9 (25.2–30.7)	0.64
3+ births	2,183	39.7 (37.5–42.0)	745	45.5 (42.3–48.6)	0.004	1,798	39.1 (37.1–41.2)	673	43.4 (39.9–46.9)	0.04
Two or more FLW visits in last trimester					0.20					0.01
No	3,620	63.2 (60.5–65.8)	1,168	66.8 (61.8–71.6)		3,287	70.8 (68.4–73.1)	997	62.3 (56.5–67.8)	
Yes	1,883	36.8 (34.2–39.5)	520	33.2 (28.4–38.2)		1,315	29.2 (26.9–31.6)	544	37.7 (32.2–43.5)	
**Gender equity**										
Age at marriage					0.64					<0.0001
<18	2,418	45.7 (42.8–48.7)	761	44.3 (39.3–49.5)		1,911	42.0 (39.3–44.7)	788	51.3 (46.9–55.7)	
18+	3,085	54.3 (51.3–57.2)	927	55.7 (50.5–60.7)		2,691	58.0 (55.3–60.7)	753	48.7 (44.3–53.1)	
Decision-making control					0.08					<0.0001
Included in all three decisions	2,316	41.5 (38.4–44.6)	556	36.2 (31.2–41.4)		2,236	48.4 (45.6–51.3)	571	36.9 (33.7–40.2)	
Excluded from at least one decision	3,187	58.5 (55.4–61.6)	1,132	63.8 (58.6–68.8)		2,366	51.6 (48.7–54.4)	970	63.1 (59.8–66.3)	
Mobility					0.18					0.99
Able to go alone to all three locations	1,056	19.4 (17.4–21.5)	244	16.6 (13.3–20.4)		635	14.0 (12.2–15.9)	202	14.0 (10.9–17.7)	
Limited mobility to at least one location	4,447	80.6 (78.5–82.6)	1,444	83.4 (79.6–86.7)		3,967	86.0 (84.1–87.8)	1,339	86.0 (82.3–89.1)	

P-values are based on survey-adjusted unpaired, two-sample t-tests.

The mean number of services/behaviors used along the RMNH CoC was significantly higher in Ananya vs. non-Ananya areas at both baseline (3.2 vs. 3.0, p = 0.01) and follow-up (4.1 vs. 3.5, p<0.0001) (Table 2). The increase over time in Ananya areas was significantly larger than that seen in non-Ananya areas (0.94 vs. 0.51 health services/behaviors, p<0.0001). Very low and very high co-coverage scores were rare. Across groups (Ananya and non-Ananya assessed at baseline and follow-up), 1–3% of women reported zero CoC components (and this decreased from baseline to follow-up), 0–4% of women reported seven or eight CoC components, and no women reported all nine CoC components. The most commonly reported CoC components at both baseline and follow-up were skilled birth attendance (63%-76%) and exclusive breastfeeding (59%-80%) (Table 3). The least commonly reported CoC components were four or more antenatal care visits (13%-24%), postnatal care (11%-16%) and postpartum contraception (10%-19%). Coverage of individual components was similar across Ananya and non-Ananya areas at baseline with the exception of delay of first bath (55.1% in Ananya areas vs. 47.2% in non-Ananya areas, p = 0.003). At midline, coverage in Ananya areas was significantly higher for four or more antenatal care visits (24.2% vs. 16.9%, p<0.0001), clean cord care (33.3% vs. 24.0%, p = 0.001), skin-to-skin care (44.1% vs. 32.7%, p<0.0001), delay of first bath (67.0% vs. 55.8%, p<0.0001) and postpartum contraceptive use (19.2% vs. 11.3%, p<0.0001). After accounting for secular trends and prior to adjusting for covariates, there were significant increases in clean cord care (OR = 1.54, p = 0.01), skin-to-skin care (OR = 1.62, p = 0.03) and postpartum contraceptive use (OR = 2.27, p<0.01) attributable to the Ananya program.

**Table 2:** Reproductive, maternal and newborn health continuum of care co-coverage. (n = 13,334).

	Baseline (n = 7,191)					Follow-up (n = 6,143)				
	Non-Ananya		Ananya			Non-Ananya		Ananya		
	Unwtd. N	% (95% CI)	Unwtd. N	% (95% CI)	p-value	Unwtd. N	% (95% CI)	Unwtd. N	% (95% CI)	p-value
0	153	2.5 (2.0–3.2)	28	1.4 (0.8–2.4)		42	1.2 (0.8–1.8)	14	0.6 (0.3–1.2)	
1	627	11.7 (10.4–13.0)	144	7.1 (5.6–9.0)		277	6.1 (5.1–7.2)	62	3.4 (2.4–4.8)	
2	1,193	21.1 (19.6–23.2)	350	19.5 (16.5–22.9)		736	14.9 (13.4–16.4)	150	9.2 (7.4–11.5)	
3	1,549	28.4 (26.4–30.5)	519	33.5 (30.0–37.1)		1,256	28.1 (26.3–29.9)	297	20.4 (17.5–23.7)	
4	1,268	23.2 (21.5–24.9)	431	25.3 (22.3–28.6)		1,213	26.9 (24.7–29.1)	434	27.3 (24.5–30.2)	
5	537	10.3 (9.0–11.8)	186	11.1 (8.7–14.0)		735	15.3 (13.7–17.1)	339	22.3 (19.3–25.6)	
6	135	2.4 (1.9–3.1)	23	1.6 (0.9–2.9)		271	6.0 (5.1–7.2)	163	11.7 (9.0–15.1)	
7	19	0.3 (0.1–0.5)	6	0.5 (0.2–1.5)		66	1.6 (1.1–2.3)	67	4.4 (3.2–6.1)	
8	2	0.0 (0.0–0.1)	1	0.0 (0.0–0.3)		6	0.1 (0.0–0.2)	15	0.6 (0.3–1.4)	
9	0	-	0	-		0	-	0	-	
Mean (95% CI)	-	3.0 (2.9–3.1)	-	3.2 (3.1–3.3)	0.01	-	3.5 (3.4–3.6)	-	4.1 (4.0–4.2)	<0.0001

P-values are based on survey-adjusted unpaired, two-sample t-tests.

**Table 3:** Reproductive, maternal and newborn health continuum of care components (n = 13,334).

	Baseline (n = 7,191)					Follow-up (n = 6,143)					Difference in differences^a^	
	Non-Ananya		Ananya			Non-Ananya		Ananya				
	Unwtd. N	% (95% CI)	Unwtd. N	% (95% CI)	p-value	Unwtd. N	% (95% CI)	Unwtd. N	% (95% CI)	p-value	OR (95% CI)	p-value
≥4 Antenatal care visits					0.14					<0.0001		
No	4,729	86.6 (84.7–88.4)	1,421	83.7 (79.9–86.8)		3,789	83.1 (81.2–84.8)	1,165	75.8 (72.1–79.1)		1.24 (0.88–1.74)	0.23
Yes	774	13.4 (11.6–15.3)	267	16.3 (13.2–20.1)		813	16.9 (15.2–18.8)	376	24.2 (20.9–27.9)			
Skilled attendant at delivery					0.09					0.13		
No	1,967	36.7 (33.9–39.5)	570	32.1 (27.7–36.8)		1,286	28.2 (25.7–30.9)	376	24.3 (20.4–28.8)		1.00 (0.75–1.32)	0.98
Yes	3,536	63.3 (60.5–66.1)	1,118	67.9 (63.2–72.3)		3,316	71.8 (69.1–74.3)	1,165	75.7 (71.2–79.6)			
Nothing applied to cord and umbilicus					0.86					0.001		
No	4,223	75.5 (73.2–77.3)	1,245	75.1 (70.8–79.0)		3,558	76.0 (73.6–78.2)	1,044	66.7 (61.8–71.2)		1.54 (1.11–2.16)	0.01
Yes	1,280	24.5 (22.3–26.8)	443	24.9 (21.0–29.2)		1,044	24.0 (21.8–26.4)	497	33.3 (28.8–38.2)			
Skin-to-skin care					0.99					<0.0001		
No	4,460	81.2 (78.8–83.4)	1,393	81.2 (75.7–85.8)		3,160	67.3 (64.2–70.3)	920	55.9 (51.2–60.6)		1.62 (1.06–2.48)	0.03
Yes	1,043	18.8 (16.6–21.2)	295	18.8 (14.4–24.3)		1,442	32.7 (29.7–35.8)	621	44.1 (39.4–48.8)			
First bath delayed by two or more days					0.003					<0.0001		
No	2,873	52.8 (50.1–55.6)	751	44.9 (40.4–49.4)		1,987	44.2 (41.5–46.9)	520	33.0 (29.3–36.9)		1.17 (0.89–1.53)	0.26
Yes	2,630	47.2 (44.4–49.9)	937	55.1 (50.6–59.6)		2,615	55.8 (53.1–58.5)	1,021	67.0 (63.1–70.7)			
Breastfed child within one hour of birth					0.69					0.09		
No	2,874	51.2 (48.5–53.9)	894	50.1 (45.2–55.0)		2,458	53.4 (50.9–55.9)	750	49.1 (44.9–53.4)		1.13 (0.85–1.51)	0.39
Yes	2,629	48.8 (46.1–51.5)	794	49.9 (45.0–54.8)		2,144	46.6 (44.1–49.1)	791	50.9 (46.6–55.1)			
Postnatal visit for mother or baby within 48 hours					0.12					0.17		
No	4,935	88.8 (87.1–90.3)	1,489	85.6 (81.4–88.9)		4,039	86.5 (84.6–88.1)	1,277	84.0 (80.8–86.8)		0.91 (0.58–1.44)	0.69
Yes	568	11.2 (9.7–12.9)	199	14.4 (11.1–18.6)		563	13.5 (11.9–15.4)	264	16.0 (13.2–19.2)			
Child exclusively breastfed					0.50					0.69		
No	2,174	39.1 (36.9–41.3)	668	40.8 (36.4–45.3)		920	21.0 (19.3–22.9)	323	20.3 (17.5–23.5)		1.12 (0.82–1.53)	0.47
Yes	3,329	60.9 (58.7–63.1)	1,020	59.2 (54.7–63.6)		3,682	79.0 (77.1–80.7)	1,218	79.7 (76.5–82.5)			
Postpartum contraception					0.21					<0.0001		
No	4,816	88.2 (86.6–89.6)	1,518	90.1 (87.2–92.4)		4,041	88.7 (87.2–90.0)	1,243	80.8 (77.6–83.6)		2.27 (1.53–3.36)	<0.01
Yes	687	11.8 (10.4–13.4)	170	9.9 (7.6–12.8)		561	11.3 (10.0–12.8)	298	19.2 (16.4–22.4)			

P-values are based on survey-adjusted unpaired, two-sample t-tests.

^a^ Adjusted for survey design, time and program.

After adjusting for covariates, the Ananya program increased overall RMNH CoC co-coverage in this sample by 0.41 (p<0.001)) health services/behaviors (Table 4). Across the multiple measures of social and gender equity assessed, eight factors retained a strong impact on CoC co-coverage even after accounting for the impact of the Ananya program–wealth, education of both the respondent and her husband, religion/caste, parity, FLW visits in the last trimester of pregnancy, respondent’s age at marriage and decision-making control. Women in the lowest wealth quartile reported 0.14 fewer health services/behaviors than women in the wealthiest quartile (p = 0.04). Women with no education, or only primary education, had significantly decreased RMNH CoC co-coverage compared with women with at least secondary education (no education coefficient = -0.24, p<0.001; primary education coefficient = -0.13, p = 0.02). Similarly, women whose husbands had no education reported 0.13 fewer health services/behaviors than women with husbands educated at the secondary or higher level (p = 0.02). Identifying as a scheduled caste or scheduled tribe, or Muslim, was also associated with decreased RMNH CoC co-coverage (SC/ST coefficient = -0.18, p<0.001; Muslim coefficient = -0.24, p<0.001). There was slightly decreased RMNH CoC co-coverage for women whose index child was their second birth, compared with primiparous mothers (coefficient = -0.08, p = 0.04). FLW visits in the final trimester of pregnancy strongly affected RMNH CoC co-coverage, with women who received fewer than two FLW visits having 0.32 fewer health services/behaviors than those who received at least two FLW visits (p<0.001). Age at marriage was a significant determinant of RMNH CoC co-coverage, with women married as children reporting receipt of 0.18 fewer CoC components than their counterparts married at or after 18 years of age (p<0.001). In contrast, women who were excluded from at least one of the three assessed decisions reported 0.13 more RMNH services/health behaviors than their counterparts who were included in all three decisions (p = 0.001).

**Table 4:** Multivariate assessment of the effect of the Ananya program on the RMNH CoC in Bihar, India (n = 13,334).

	Coefficient (95% CI)	p-value
**Ananya program effect (program x time)**	**0.41 (0.24–0.59)**	**<0.001**
**Demographic and social equity**		
Age at interview		
15–19	-0.09 (-0.32–0.14)	0.45
20–24	-0.08 (-0.25–0.09)	0.36
25–29	-0.08 (-0.26–0.09)	0.37
30–34	-0.10 (-0.27–0.06)	0.22
35+	REF	
Wealth quartile		
1 (lowest)	**-0.14 (-0.28- -0.01)**	**0.04**
2	-0.12 (-0.26–0.01)	0.07
3	-0.05 (-0.16–0.05)	0.31
4 (highest)	REF	
Education		
None	**-0.24 (-0.36- -0.12)**	**<0.001**
Primary	**-0.13 (-0.24- -0.02)**	**0.02**
Secondary	REF	
Husband’s education		
None	**-0.13 (-0.23- -0.02)**	**0.02**
Primary	-0.12 (-0.22- -0.03)	0.01
Secondary	REF	
Scheduled caste/scheduled tribe / Muslim		
SC/ST only	**-0.18 (-0.27- -0.09)**	**<0.001**
Muslim only	**-0.24 (-0.36- -0.12)**	**<0.001**
Not SC/ST or Muslim	REF	
Gender of focal child		
Male	REF	
Female	-0.02 (-0.09–0.06)	0.66
Parity		
1 birth	REF	
2 births	**-0.08 (-0.16- -0.003)**	**0.04**
3+ births	-0.04 (-0.13–0.05)	0.38
Two or more FLW visits in last trimester		
No	**-0.32 (-0.40- -0.25)**	**<0.001**
Yes	REF	
**Gender equity**		
Age at marriage		
<18	**-0.18 (-0.26- -0.09)**	**<0.001**
≥18	REF	
Decision-making control		
Included in all three decisions	REF	
Excluded from at least one decision	**0.13 (0.05–0.20)**	**0.001**
Mobility		
Able to go alone to all three locations	REF	
Limited mobility to at least one location	-0.05 (-0.15–0.05)	0.33

Linear regression model adjusts for survey design and weights, program and time.

In addition to assessing Ananya’s effect on RMNH CoC co-coverage, program effect was assessed for each of the nine health services/behaviors included in the overall co-coverage metric. Ananya increased the odds of clean cord care by 72% (p<0.01), of skin-to-skin care by 59% (p = 0.03) and of postpartum contraceptive use by 130% (p<0.001)(Table 5; full models in S1 Table). There was no significant, attributable program effect on antenatal care, skilled birth attendance, delayed first bath, early initiation of breastfeeding, postnatal care within two days or exclusive breastfeeding.

**Table 5:** Overall Ananya program effect on RMNH CoC and components, and the influence of gender equity measures on program effect (n = 13,334).

	≥4 Antenatal care visits^a^	Skilled attendant at delivery^a^	Nothing applied to cord and umbilicus^a^	Skin-to-skin care^a^	First bath delayed by two or more days^a^	Breasted child within one hour of birth^a^	Postnatal visit for mother or baby within 48 hours^a^	Child exclusively breastfed^a^	Postpartum contraception^a^	RMNH CoC co-coverage (0–9) ^b^
Ananya program effect (program x time)^c^	1.27 (0.89–1.82)	1.03 (0.76–1.38)	1.72 (1.23–2.40)**	1.59 (1.04–2.42)*	1.18 (0.90–1.54)	1.15 (0.87–1.53)	0.72 (0.46–1.14)	1.13 (0.83–1.54)	2.30 (1.54–3.45)***	0.41 (0.24–0.59)***
Influence of marriage at <18 years on program effect^d^	0.36 (0.18–0.71)**^e^	0.61 (0.37–0.99)*^e^	1.08 (0.62–1.89)^e^	0.75 (0.36–1.58)^e^	1.54 (0.89–2.64)^e^	1.34 (0.83–2.16)^e^	0.90 (0.38–2.14)^e^	0.56 (0.35–0.90)*^e^	1.00 (0.48–2.09) ^e^	-0.27 (-0.55–0.01)^f^
Ananya program effect: married <18	0.69 (0.40–1.18)	0.81 (0.54–1.22)	1.65 (1.10–2.47)*	1.37 (0.72–2.62)	1.47 (1.00–2.15)*	1.38 (0.96–1.98)	0.72 (0.41–1.25)	0.82 (0.53–1.26)	2.25 (1.35–3.75)**	0.27 (0.04–0.50)*
Ananya program effect: married ≥18	1.91 (1.22–3.00)**	1.34 (0.94–1.91)	1.79 (1.12–2.86)*	1.82 (1.12–2.95)*	0.96 (0.65–1.41)	1.03 (0.70–1.51)	0.80 (0.39–1.63)	1.45 (1.03–2.05)*	2.25 (1.26–4.00)**	0.54 (0.32–0.76)***
Influence of limited decision-making control on program effect^g^	1.12 (0.54–2.31)^h^	1.39 (0.86–2.26)^h^	0.65 (0.37–1.15)^h^	1.10 (0.53–2.31)^h^	0.78 (0.44–1.36^h^	1.04 (0.60–1.82)^h^	2.09 (0.99–4.39)^h^	0.93 (0.52–1.66) ^h^	0.74 (0.37–1.49) ^h^	0.07 (-0.26–0.40)^i^
Ananya program effect: excluded from at least one decision	1.42 (0.91–2.21)	1.17 (0.82–1.68)	1.42 (0.94–2.15)	1.69 (1.07–2.67)*	1.09 (0.79–1.50)	1.13 (0.80–1.60)	0.98 (0.56–1.71)	1.07 (0.75–1.53)	2.01 (1.23–3.29)**	0.43 (0.22–0.65)***
Ananya program effect: included in all three decisions	1.27 (0.70–2.30)	0.84 (0.55–1.28)	2.18 (1.36–3.50)**	1.53 (0.75–3.12)	1.41 (0.88–2.26)	1.08 (0.67–1.73)	0.47 (0.25–0.86)*	1.15 (0.69–1.90)	2.71 (1.52–4.86)**	0.36 (0.08–0.64)*
Influence of limited mobility on program effect^j^	1.16 (0.46–2.92)^k^	1.23 (0.63–2.39)^k^	0.72 (0.29–1.78)^k^	1.06 (0.56–2.04)^k^	0.57 (0.27–1.19)^k^	0.92 (0.45–1.88)^k^	1.86 (0.63–5.47)^k^	0.65 (0.28–1.49)^k^	1.00 (0.40–2.51)^k^	-0.15 (-0.60–0.30)^l^
Ananya program effect: limited mobility to at least one location	1.30 (0.89–1.90)	1.06 (0.76–1.47)	1.64 (1.14–2.35)**	1.63 (1.07–2.48)*	1.07 (0.82–1.40)	1.15 (0.85–1.55)	0.78 (0.48–1.28)	1.06 (0.77–1.46)	2.34 (1.47–3.72)***	0.39 (0.20–0.58)***
Ananya program effect: able to go alone to all three locations	1.12 (0.47–2.67)	0.86 (0.47–1.56)	2.29 (0.98–5.38)	1.53 (0.74–3.17)	1.88 (0.92–3.87)	1.24 (0.64–2.41)	0.42 0.15–1.16)	1.63 (0.74–3.58)	2.34 (1.04–5.24)*	0.54 (0.11–0.96)*

Estimates presented are program effect estimators (95% CI). Logistic models show adjusted odds ratios, linear models show adjusted linear regression coefficients.

*p<0.05;

**p<0.01;

***p<0.001

^a^ Logistic regression model

^b^ Linear regression model.

^c^ Models adjust for all variables shown in Table 3.

^d^ Three-way interaction term shown. Models adjust for all variables shown in Table 3 as well as a three-way interaction between time, treatment and age at marriage.

^e^ Ratio of Ananya program effect for women married <18 to those married ≥18.

^f^ Difference in Ananya program effect between women married at <18 and women married at ≥18.

^g^ Three-way interaction term shown. Limited decision-making control indicates exclusion from at least one decision. Models adjust for all variables shown in Table 3 as well as a three-way interaction between time, treatment and decision-making control.

^h^ Ratio of Ananya program effect for women excluded from at least one decision to women included in all three decisions.

^i^ Difference in Ananya program effect between women excluded from at least one decision and women included in all three decisions.

^j^ Three-way interaction term shown. Limited mobility indicates limited mobility to at least one location. Models adjust for all variables shown in Table 3 as well as a three-way interaction between time, treatment and mobility.

^k^ Ratio of Ananya program effect for women with limited mobility to at least one location to women able to go to all three locations.

^l^ Difference in Ananya program effect between women with limited mobility to at least one location and women able to go to all three locations.

Finally, analyses were conducted to further explore the influence of gender equity measures (age at marriage, decision-making control and limited mobility) on Ananya’s program effect on CoC co-coverage. Child marriage compromised Ananya’s program effect on antenatal care, skilled birth attendance and exclusive breastfeeding. Specifically, the program effect on four or more antenatal care visits among women married as adults was reduced by a factor of 0.36 for women married as children (aOR = 1.91 vs. aOR = 0.69; p = 0.003) (Table 5). Ananya program effect on having a skilled attendant at delivery among women married as adults was reduced by factor of 0.61 for women married as children (aOR = 1.34 vs. aOR = 0.81; p = 0.049), and program effect on exclusive breastfeeding among women married as adults was reduced by a factor of 0.56 for women married as children (aOR = 1.45 vs. aR = 0.82; p = 0.02). Limitations on decision-making and mobility did not influence the effect of the Ananya program on CoC co-coverage or any of the nine CoC component health services/behaviors. To further explore whether observed child marriage effects were an artifact of effects for the younger mothers in the sample, we also conducted a three-way interaction analysis with age of mother, and findings were not significant (results not shown).

## Discussion

The Ananya program, through its supply- and demand-side interventions for maternal and newborn health, significantly increased RMNH CoC co-coverage among this sample of recent mothers and infants in Bihar. The 0.41 gain in health services/behaviors represents a 13% improvement over baseline attributable to the Ananya program, and is important both because each intervention along this continuum was selected for its demonstrated importance in improving maternal and newborn health and survival, and because this achievement was realized in a population with substantial social and gender inequities. Improving CoC co-coverage is particularly important in this context given its very low level, with less than 5% of participants reporting six or more of the nine assessed behaviors/services at baseline.

Despite program implementation, no recent mothers in this sample reported all nine services/behaviors measured, and very few reported seven or eight of the assessed components. Coverage of many of these interventions was low at the inception of the Ananya program–four of the assessed services/behaviors had coverage below 20% at baseline—and thus the challenges of sustained service utilization/behavior were substantial. The health services/behaviors that make up the RMNH CoC in this study can be broadly grouped into two categories: health service encounters (antenatal care, skilled birth attendance and postnatal care) and health behaviors (i.e. clean cord care, skin-to-skin care, delayed first bath, early initiation of breastfeeding, exclusive breastfeeding and postpartum contraceptive use), the latter of which could be facilitated by FLWs or other providers. The increase in co-coverage was primarily driven by significant improvements in health behaviors: clean cord care, skin-to-skin care and postpartum contraceptive use. The 2.3 times increased odds of postpartum contraceptive use is particularly striking given that both baseline and follow-up samples were comprised of mothers of 0–5 month olds (and lactational amenorrhea was excluded from our measure of postpartum contraceptive use). These are key interventions—clean cord care can reduce neonatal mortality by approximately 37%, and skin-to-skin care has been associated with neonatal survival in this and similar populations, and can reduce neonatal mortality among premature newborns by 30–50% [31–33]. Modern contraceptive use is associated with antenatal care acquisition, childhood immunization and maternal and neonatal mortality [34, 35]. When assessed individually, there were no negative associations between low education levels (for the respondent or her husband) and clean cord care, skin-to-skin care or postpartum contraception, indicating that a key social inequity may have been overcome for these outcomes in this context, though wealth, caste/religion and gender equity were still compromised.

In terms of health encounters, findings suggest that Ananya requires some alteration to better support best practices among these recent mothers. None of the three encounter-based outcomes (antenatal care, skilled birth attendance and postnatal care) increased in this sample based on receipt of this program. The primary factors associated with decreased utilization of these encounters were social equity measures such as wealth, education and religion/caste. Additional outreach to these marginalized groups may be necessary to improve utilization, a suggestion supported by the strongly protective association of FLW visits in the last trimester with all three health encounter outcomes. Improved quality of care in this context may also support better utilization, as previous research has documented that poor quality care in Bihar keeps individuals from seeking services [17, 33, 36, 37]. This is a reasonable response given that quality issues such as inadequate numbers of providers, poor hygiene in care practices, and mistreatment by providers are well documented in Bihar and India and such quality issues are associated with increased maternal and child health outcomes and even death [38–41].

The implications of this lack of effect on health encounters in this sample are concerning, as from a continuum perspective, sustained linkages across all three services have demonstrated benefit for reducing neonatal mortality [14]. Individually, beyond its benefits for mortality and morbidity reduction [6, 42, 43], antenatal care is an important area for initial contact with regards to continuation through the continuum of care, in this population and others [33, 42]. Skilled birth attendance can improve maternal survival, and reduce stillbirths and neonatal mortality (by 23–45% and 11–17%, respectively) [6, 26, 44]. Postnatal care is a critical encounter in which to provide care and education, with the potential to reduce neonatal mortality by 30–60%, an association also seen in Bihar [33, 45–49]. As institutional deliveries and skilled birth attendance have more than doubled over the past decade, women who are still not using institutional deliveries and skilled birth attendance may have greater utilization barriers than women who have shifted their delivery practices since the initiation of the Government of India’s JSY initiative, which involves cash transfer for facility delivery [50]. Similar cash transfer approaches may offer an opportunity to quickly increase coverage of four or more antenatal care visits, and postnatal care within two days, but sustainability considerations are key. It is also important to note that the three health encounter metrics assess the presence/quantity of these encounters, rather than the quality of services received therein, and as noted above, service may be key to helping promote use of these services [38–41].

An additional key finding of this study is the beneficial effect of FLW visits during pregnancy for these recent mothers, which were strongly and positively associated with increased RMNH CoC co-coverage overall, and for the component outcomes of antenatal care, skilled birth attendance, early initiation of breastfeeding, postnatal care visits within two days, exclusive breastfeeding and postpartum contraceptive use, above and beyond the benefit seen from the FLW-focused aspects of the Ananya program. This finding builds on prior work demonstrating the utility of trained and supported FLWs, in particular community health workers, to help mobilize communities to engage in healthier neonatal and postnatal health practices [25, 51–55]. FLWs may also be key to facilitate linkages across the place component of the continuum of care, connecting interventions delivered at home, in the community and in health facilities–the importance of this role, and its association with reduced neonatal and maternal mortality, has been demonstrated elsewhere [14, 54], and merits greater focus in this setting.

Despite these promising findings regarding Ananya impact for recent mothers, program effects in this sample appear to be compromised by gender inequity, specifically child marriage. The benefits of the Ananya program were not strong enough to neutralize the depressed co-coverage scores of recent mothers who married as minors, as well as those who were poor, who had low education levels or husbands with low education, higher parity or were of marginalized caste/religion (all factors related to child marriage) [18]. Additionally, moderation analyses indicate a significant interaction of child marriage on program impact, with a lesser effect of the Ananya program on antenatal care, skilled birth attendance and exclusive breastfeeding specifically, for recent mothers married as minors compared to those married as adults. These findings suggest that Ananya and programs like it would benefit from more focused efforts for women married as minors. Exploratory analyses indicating that age of mother did not moderate impact where child marriage did suggests that youth-focused programming alone will not address this issue. Screening for this history may be an important element of service tailoring to better meet the needs of this population. Larger structural changes preventing child marriage and supporting greater gender equity for women would also be beneficial, and such an approach has been recommended more broadly for maternal and child health programming [56, 57].

Less clear from the current analyses are findings related to women’s decision-making control and CoC utilization, which indicated that women in this sample with limited decision-making agency used significantly more RMNH CoC services/behaviors than their counterparts with greater decision-making control, though that did not significantly influence the Ananya program effect for any assessed outcome. These findings may be an issue of the measure used, as there are some concerns with the validity of this measure across national contexts [58]. However, it may also be that decision-making control does not impede health care utilization if others in greater control are supportive of evidence-based RMNH practices, or even compel use of some services, measures which were not assessed in these data. An additional possible explanation is that quality of care is low for facility-based services provided in Bihar (also not comprehensively assessed within this data, but indicated elsewhere [17, 33]), and that women with greater agency would be more discerning about where and from whom they sought care.

This analysis has several limitations. The study design is quasi-experimental, not randomized, allowing for potential differences between groups not otherwise considered; comparability on demographics and behaviors at baseline alleviate some but not all of this concern. Inability to measure outcomes over time for the same participants, and lack of clarity regarding quality and intensity of the Ananya program exposure, impedes full understanding of program impact. All data used are based on women’s self-report, and therefore susceptible to recall and social desirability bias, though the former is lessened by the fact that questions focused on the most recent pregnancy, which occurred within the six months prior to interview. Finally, findings are limited to Bihar, and cannot be assumed to be generalizable to other states within India or other countries, though findings may be more generalizable to very low resource contexts like Bihar, where disproportionate burden of maternal and neonatal concerns exist.

### Conclusion

Our findings indicate that the Ananya program was effective at improving RMNH CoC co-coverage among recent mothers. Gains were largely due to improved health behaviors (clean cord care, skin-to-skin care and postpartum contraception) rather than health care utilization (i.e., antenatal care, skilled birth attendance). Child marriage further compromised program effect on health care utilization, as well as exclusive breastfeeding. These findings suggest that supporting the public health system with focused RMNCH training and community outreach interventions can improve RMNH CoC co-coverage. However, such programs may benefit from screening women for age at marriage and providing additional supports for those married as minors. Additionally, more work is needed to support improvements to CoC co-coverage inclusive of health care service utilization; such efforts likely require improved quality of care as well as linkage to care in low resource settings such as Bihar.

## Supporting information

S1 Table Multivariate assessment of the effect of the Ananya program on RMNH CoC components in Bihar, India (n = 13,334). (DOCX)
