# Supplementary material for: Making the continuum of care work for mothers and infants: Does gender equity matter? Findings from a quasi-experimental study in Bihar, India
Source: PLoS One. 2017 Feb 1;12(2):e0171002. doi: 10.1371/journal.pone.0171002 (PMC5287473; doi:10.1371/journal.pone.0171002)
Supplement: S1 Table — (DOCX) [file pone.0171002.s001.docx]

**S1 Table. Multivariate assessment of the effect of the Ananya program on RMNH CoC components in Bihar, India (n=13,334).**

|  | ≥4 Antenatal care visits | Skilled attendant at delivery | Nothing applied to cord and umbilicus | Skin-to-skin care | First bath delayed by two or more days | Breasted child within one hour of birth | Postnatal visit for mother or baby within 48 hours | Child exclusively breastfed | Postpartum contraception |
| --- | --- | --- | --- | --- | --- | --- | --- | --- | --- |
| **Ananya program effect (program x time)** | - 1.27 - (0.89-1.82) | - 1.03 - (0.76-1.38) | - 1.72 - (1.23-2.40)** | - 1.59 - (1.04-2.42)* | - 1.18 - (0.90-1.54) | - 1.15 - (0.87-1.53) | - 0.72 - (0.46-1.14) | - 1.13 - (0.83-1.54) | - 2.30 - (1.54-3.45)*** |
| **Demographic and social equity** |  |  |  |  |  |  |  |  |  |
| Age at interview |  |  |  |  |  |  |  |  |  |
| - 15-19 | - 1.08 - (0.66-1.78) | - 0.73 - (0.48-1.09) | - 0.90 - (0.57-1.42) | - 0.96 - (0.58-1.59) | - 0.83 - (0.56-1.22) | - 0.88 - (0.60-1.29) | - 1.71 - (0.95-3.06) | - 1.72 - (1.12-2.61)* | - 0.45 - (0.24-0.86)* |
| - 20-24 | - 0.91 - (0.61-1.38) | - 0.90 - (0.68-1.20) | - 0.96 - (0.71-1.31) | - 0.97 - (0.71-1.33) | - 0.81 - (0.62-1.06) | - 0.99 - (0.76-1.29) | - 1.13 - (0.76-1.68) | - 1.72 - (1.31-2.26)*** | - 0.45 - (0.31-0.64)*** |
| - 25-29 | - 1.07 - (0.73-1.57) | - 0.87 - (0.66-1.14) | - 0.98 - (0.74-1.30) | - 0.92 - (0.68-1.24) | - 0.76 - (0.59-0.98)* | - 1.08 - (0.84-1.39) | - 1.04 - (0.74-1.45) | - 1.58 - (1.20-2.08)** | - 0.50 - (0.37-0.68)*** |
| - 30-34 | - 1.37 - (0.90-2.07) | - 0.95 - (0.72-1.26) | - 0.89 - (0.65-1.22) | - 1.02 - (0.75-1.38) | - 0.68 - (0.53-0.87)** | - 0.96 - (0.74-1.24) | - 1.26 - (0.88-1.80) | - 1.27 - (0.96-1.68) | - 0.57 - (0.39-0.82)** |
| - 35+ | REF | REF | REF | REF | REF | REF | REF | REF | REF |
| Wealth quartile |  |  |  |  |  |  |  |  |  |
| - 1 (lowest) | - 0.45 - (0.34-0.58)*** | - 0.57 - (0.45-0.70)*** | - 1.99 - (1.62-2.45)*** | - 0.57 - (0.45-0.71)*** | - 1.50 - (1.24-1.81)*** | - 1.08 - (0.90-1.29) | - 0.62 - (0.47-0.82)** | - 1.36 - (1.11-1.68)** | - 0.42 - (0.32-0.55)*** |
| - 2 | - 0.52 - (0.42-0.65)*** | - 0.63 - (0.53-0.76)*** | - 1.35 - (1.11-1.63)** | - 0.91 - (0.73-1.13) | - 1.29 - (1.08-1.53)** | - 1.04 - (0.86-1.25) | - 0.88 - (0.67-1.15) | - 1.22 - (1.00-1.49)* | - 0.48 - (0.37-0.62)*** |
| - 3 | - 0.59 - (0.48-0.72)*** | - 0.80 - (0.66-0.98)* | - 1.20 - (0.97-1.48) | - 0.84 - (0.70-1.01) | - 1.10 - (0.95-1.28) | - 1.04 - (0.90-1.20) | - 1.06 - (0.83-1.36) | - 1.29 - (1.07-1.55)** | - 0.79 - (0.64-0.97)* |
| - 4 (highest) | REF | REF | REF | REF | REF | REF | REF | REF | REF |
| Education |  |  |  |  |  |  |  |  |  |
| - None | - 0.43 - (0.33-0.56)*** | - 0.57 - (0.46-0.70)*** | - 1.04 - (0.85-1.28) | - 1.15 - (0.95-1.39) | - 0.80 - (0.68-0.95)* | - 0.94 - (0.78-1.13) | - 1.15 - (0.86-1.54) | - 1.07 - (0.89-1.29) | - 1.00 - (0.75-1.33) |
| - Primary | - 0.59 - (0.45-0.76)*** | - 0.78 - (0.63-0.96)* | - 1.00 - (0.82-1.23) | - 1.02 - (0.83-1.25) | - 0.91 - (0.77-1.07) | - 1.03 - (0.87-1.21) | - 1.17 - (0.90-1.51) | - 1.03 - (0.86-1.23) | - 0.99 - (0.78-1.25) |
| - Secondary | REF | REF | REF | REF | REF | REF | REF | REF | REF |
| Husband’s education |  |  |  |  |  |  |  |  |  |
| - None | - 0.77 - (0.60-0.99)* | - 0.74 - (0.63-0.88)** | - 1.06 - (0.86-1.29) | - 0.98 - (0.80-1.20) | - 0.87 - (0.74-1.03) | - 0.97 - (0.81-1.15) | - 1.19 - (0.92-1.53) | - 0.93 - (0.78-1.10) | - 0.92 - (0.71-1.19) |
| - Primary | - 0.84 - (0.69-1.02) | - 0.75 - (0.65-0.87)*** | - 1.04 - (0.88-1.22) | - 0.98 - (0.83-1.16) | - 0.94 - (0.80-1.09) | - 0.99 - (0.86-1.15) | - 0.98 - (0.75-1.27) | - 0.91 - (0.78-1.07) | - 0.92 - (0.74-1.15) |
| - Secondary | REF | REF | REF | REF | REF | REF | REF | REF | REF |
| Scheduled caste/scheduled tribe / Muslim |  |  |  |  |  |  |  |  |  |
| - SC/ST only | - 0.67 - (0.56-0.81)*** | - 0.74 - (0.63-0.87)*** | - 0.78 - (0.65-0.92)** | - 1.14 - (0.95-1.37) | - 0.81 - (0.70-0.94)** | - 1.02 - (0.88-1.19) | - 1.06 - (0.85-1.33) | - 0.90 - (0.78-1.03) | - 1.12 - (0.92-1.36) |
| - Muslim only | - 0.90 - (0.71-1.13) | - 0.61 - (0.50-0.76)*** | - 0.91 - (0.74-1.12) | - 0.82 - (0.63-1.05) | - 0.92 - (0.77-1.11) | - 0.87 - (0.72-1.06) | - 0.75 - (0.60-0.95)* | - 1.27 - (1.04-1.55)* | - 0.62 - (0.48-0.80)*** |
| - Not SC/ST or Muslim | REF | REF | REF | REF | REF | REF | REF | REF | REF |
| Gender of focal child |  |  |  |  |  |  |  |  |  |
| - Male | REF | REF | REF | REF | REF | REF | REF | REF | REF |
| - Female | - 0.99 - (0.88-1.12) | - 0.98 - (0.87-1.09) | - 1.06 - (0.95-1.19) | - 0.97 - (0.86-1.10) | - 1.00 - (0.89-1.13) | - 0.97 - (0.87-1.08) | - 0.99 - (0.83-1.18) | - 1.10 - (0.98-1.23) | - 0.74 - (0.64-0.87)*** |
| Parity |  |  |  |  |  |  |  |  |  |
| - 1 birth | REF | REF | REF | REF | REF | REF | REF | REF | REF |
| - 2 births | - 0.73 - (0.62-0.86)*** | - 0.75 - (0.64-0.89)** | - 1.12 - (0.92-1.35) | - 0.93 - (0.80-1.08) | - 0.92 - (0.79-1.08) | - 0.96 - (0.83-1.12) | - 0.99 - (0.78-.26) | - 1.03 - (0.90-1.19) | - 1.44 - (1.13-1.84)** |
| - 3+ births | - 0.61 - (0.50-0.76)*** | - 0.64 - (0.53-0.77)*** | - 1.14 - (0.92-1.41) | - 0.85 - (0.70-1.03) | - 0.91 - (0.77-1.08) | - 1.00 - (0.85-1.17) | - 1.18 - (0.93-1.50) | - 1.12 - (0.96-1.32) | - 2.87 - (2.22-3.70)*** |
| Two or more FLW visits in last trimester |  |  |  |  |  |  |  |  |  |
| - No | - 0.80 - (0.68-0.94)** | - 0.73 - (0.63-0.84)*** | - 1.71 - (1.46-2.01)*** | - 0.91 - (0.79-1.06) | - 0.89 - (0.78-1.01) | - 0.85 - (0.76-0.95)** | - 0.20 - (0.16-0.25)*** | - 0.88 - (0.78-0.998)* | - 0.78 - (0.67-0.90)** |
| - Yes | REF | REF | REF | REF | REF | REF | REF | REF | REF |
| **Gender equity** |  |  |  |  |  |  |  |  |  |
| Age at marriage |  |  |  |  |  |  |  |  |  |
| - <18 | - 0.97 - (0.83-1.13) | - 0.84 - (0.74-0.95)** | - 0.76 - (0.66-0.89)*** | - 0.87 - (0.76-0.995)* | - 0.84 - (0.74-0.96)* | - 0.84 - (0.74-0.96)* | - 1.23 - (1.00-1.51)* | - 1.05 - (0.92-1.20) | - 0.91 - (0.76-1.08) |
| - ≥18 | REF | REF | REF | REF | REF | REF | REF | REF | REF |
| Decision-making control |  |  |  |  |  |  |  |  |  |
| - Included in all three decisions | REF | REF | REF | REF | REF | REF | REF | REF | REF |
| - Excluded from at least one decision | - 1.56 - (1.31-1.86)*** | - 1.08 - (0.95-1.23) | - 1.11 - (0.97-1.28) | - 1.51 - (1.29-1.78)*** | - 1.20 - (1.08-1.35)** | - 0.75 - (0.65-0.85)*** | - 0.78 - (0.65-0.95)* | - 1.04 - (0.91-1.19) | - 1.12 - (0.95-1.33) |
| Mobility |  |  |  |  |  |  |  |  |  |
| - Able to go alone to all three locations | REF | REF | REF | REF | REF | REF | REF | REF | REF |
| - Limited mobility to at least one location | - 0.83 - (0.69-1.01) | - 0.99 - (0.84-1.16) | - 1.31 - (1.08-1.58)** | - 0.62 - (0.51-0.76)*** | - 1.10 - (0.92-1.32) | - 1.00 - (0.87-1.17) | - 0.88 - (0.68-1.14) | - 1.20 - (1.01-1.42) | - 0.73 - (0.67-0.90)** |

Logistic regression models adjust for survey design and weights, program and time. Values shown are adjusted odds ratios (95% confidence intervals).

*p<0.05; **p<0.01; ***p<0.001
